# Supplementary material for: Inhibition of DYRK1A disrupts neural lineage specificationin human pluripotent stem cells
Source: eLife. 2017 Sep 8;6:e24502. doi: 10.7554/eLife.24502 (PMC5656431; doi:10.7554/eLife.24502)
Supplement: Supplementary file 2. — The text describes general experimental procedures and details of chemical syntheses and physical properties of the novel compounds used in this study. [file elife-24502-supp2.docx]

**Supplementary File 2**

**Chemical Synthesis**

**General**

Reactions were monitored using thin layer chromatography (TLC) performed with aluminium-backed plates of Merck Silica Gel 60 F_254_. Detection was effected by charring in a mixture of ceric ammonium molybdate (CAM) stain (1 g Ce(SO_4_)_2_, 5 g (NH_4_)_6_Mo_7_O_24_.4H_2_O in 100 ml 1.2 M H_2_SO_4_) or potassium permanganate stain (1 g KMnO_4_, 6.7 g K_2_CO_3_, 1.7 ml 5% aq. NaOH in 100 ml H_2_O), and/or visualizing with UV light. DMF was dried over activated 4 Å molecular sieves. Dichloromethane was dried using a Grubbs solvent purification apparatus as described by Pangborn *et al*.[^1^](#_ENREF_1) K_2_CO_3_ was dried by flame drying with stirring under <1 mbar vacuum. ^1^H and ^13^C NMR spectra were recorded using Varian Inova-400 (399.7 MHz for ^1^H and 100.5 MHZ for ^13^C) or Varian Inova-500 (499.7 MHz for ^1^H and 125.8 MHZ for ^13^C) spectrometers. Spectra were obtained at 298 K unless stated otherwise. Residual solvent peaks were used as internal references for ^1^H NMR spectra: chloroform (δ 7.26 ppm), methanol (δ 3.31 ppm), and dimethylsulfoxide (δ 2.50 ppm). Chemical shifts (δ) are reported in parts per million (ppm) relative to internal references and splitting patterns are designated as s, singlet; br s, broad singlet; d, doublet; dd, doublet of doublets; t, triplet; q, quartet; m, multiplet. Solvent peaks were used as internal references for ^13^C NMR spectra: chloroform (δ 77.16 ppm), methanol (δ 49.00 ppm) and dimethylsulfoxide (δ 39.52 ppm). Melting points were obtained using a Reichert–Jung hotstage microscope. High resolution mass spectra (HRMS) were obtained on an Agilent 6220 LC/ESI-TOF mass spectrometer, or a Finnigan hybrid FT-ICR mass spectrometer (Thermo Electron Corp).

**2-Dimethylamino-1-nitropropene (1)**

A solution of *N,N*-dimethylacetamide dimethylacetal (400 µl, 2.74 mmol) and nitromethane (178 µl, 3.28 mmol) in ethanol (0.7 ml) was refluxed for 10 min. The solution was cooled to rt and the solvent evaporated under vacuum. Flash chromatography of the residue (EtOAc), followed by recrystallization (2-propanol) afforded the nitroenamine **1** as a yellow powder (239 mg, 67%), mp 82-85 ^o^C (lit.[^2^](#_ENREF_2) 83 ^o^C); δ_H_(500 MHz, CDCl­_3_) 6.76 (1 H, s, C=CH), 3.07 (6 H, s, N(CH_3_)_2_), 2.62 (3 H, s, C**C**H_3_); δ_C_(125 MHz, CDCl­_3_) 160.6, 113.7 (2 C, alkene), 41.0 (2 C, N(CH_3_)_2_), 16.5 (1 C, CH_3_); HRMS (ESI^+^) *m/z* 153.0635 (C_5_H_10_N_2_NaO_2_ [M + Na]^+^ requires 153.0640).

**2-(*p*-Methoxyphenylamino)-1-nitro-propene (2)**

A solution of *p*-toluenesulfonic acid (254 mg, 1.47 mmol), *p*-anisidine (544 mg, 4.42 mmol) and nitroenamine **1** (192 mg, 1.47 mmol) in DMF (0.6 ml) was stirred at rt for 2 h. The mixture was diluted with water (10 ml) and the resultant precipitate was filtered, washed with water (3 × 1 ml) and dried. The filter cake was recrystallised (2-propanol) to afford the nitroenamine **2** as olive-green crystals (185 mg, 60%), mp 84-85 ^o^C (lit.[^3^](#_ENREF_3) 85-87 ^o^C); δ_H_(500 MHz, CDCl­_3_) 11.46 (1 H, bs, NH), 6.92-7.11 (4 H, m, C_6_H_4_), 6.65 (1 H, s, C=CH), 3.83 (3 H, s, OCH_3_), 1.93 (3 H, s, ArCH_3_); δ_C_(125 MHz, CDCl­_3_) 159.1, 157.1 (2 C, C=CH), 129.4, 127.2, 114.8, 111.6 (6 C, Ar), 55.7 (1 C, OCH_3_), 18.1 (1 C, Ar**C**H_3_); HRMS (ESI^+^) *m/z* 231.0740 (C_10_H_12_N_2_NaO_3_ [M + Na]^+^ requires 231.0746).

**6-Hydroxy-3-nitro-1-(*p*-methoxyphenyl)-2-methylindole (ID-8)**

A solution of nitroenamine **2** (163 mg, 0.784 mmol) and *p*-benzoquinone (140 mg, 1.30 mmol) in acetic acid (1 ml) and acetic anhydride (0.1 ml) was stirred at rt for 48 h. A precipitate formed which was filtered and washed with acetic acid, and water. The filter cake was recrystallized (AcOH) to afford **ID-8** as yellow crystals (57 mg, 24%), mp 255-258 ^o^C (decomp.) (lit.[^3^](#_ENREF_3) 265-266 ^o^C decomp.); δ_H_(500 MHz, d­_6_-DMSO) 9.53 (1 H, bs, OH), 7.95 (1 H, d, *J*_4,5_ 8.5 Hz, H4), 7.19-7.48 (4 H, m, C_6_H_4_), 6.86 (1 H, dd, *J*_4,5_ 8.5 Hz, *J*_5,7_ 2.0 Hz, H5), 6.37 (1 H, d, *J*_5,7_ 2.0 Hz, H7), 3.87 (3 H, s, ArOC**H**_3_), 2.55 (3 H, s, CH_3_); δ_C_(125 MHz, d_6_-DMSO) 159.9, 155.4, 142.8, 137.1, 129.3, 127.1, 126.3, 120.6, 115.2, 113.8, 113.2, 96.7 (14 C, Ar), 55.6 (1 C, OCH_3_), 13.5 (1 C, Ar**C**H_3_); HRMS (ESI^+^) *m/z* 321.0846 (C_16_H_14_N_2_NaO_4_ [M + Na]^+^ requires 321.0851).

**3-Acetamido-6-acetoxy-1-(*p*-methoxyphenyl)-2-methylindole (34)**

A solution of acetic anhydride (30 µl) and **ID-8** (33 mg, 0.17 mmol) in AcOH (0.2 ml) was heated to 100 ^o^C and zinc powder (109 mg, 1.68 mmol) was added. The mixture was stirred for 2 h at reflux (125 ^o^C). The zinc powder was removed with hot filtration, and excess AcOH was removed under reduced pressure. Water (5 ml) was added to the residue and the mixture was was extracted with EtOAc (3 ×5 ml). The combined organic extracts were washed with water then brine, dried (MgSO_4_) and concentrated under reduced pressure. The crude mixture was purified with flash chromatography (20-80% EtOAc/pet. spirits) followed by recrystallization (EtOAc/pet. spirits) to afford the indole **34** as beige powder (12.4 mg, 21%), mp 199-201 ^o^C; δ_H_(500 MHz, d_6_-DMSO) 9.27 (1 H, NH), 7.13-7.35 (5 H, m, C_6_H_4_,H4), 6.78 (1 H, m, H5), 6.70 (1 H, m, H7), 3.86 (3 H, s, OCH_3_), 2.09-2.20 (9 H, 3 s, 3 × CH_3_); δ_C_(125 MHz, d_6_-DMSO) 169.8, 168.8 (2 C, C=O), 158.8, 145.9, 135.4, 131.8, 129.3, 128.9, 122.2, 118.3, 115.0, 114.0, 111.9, 102.8 (14 C, Ar), 55.5 (1 C, OCH_3_), 22.8, 20.9, 10.7 (3 C, 3 × CH_3_); HRMS (ESI^+^) *m/z* 353.1496 (C_20_H_21_N_2_O_4_ [M + H]^+^ requires 353.1495).

**6-Methoxy-1-(*p*-methoxyphenyl)-2-methyl-3-nitroindole (28)**

A solution of iodomethane (42 µl, 0.68 mmol), **ID-8** (20 mg, 0.07 mmol) and K_2_CO_3_ (19 mg, 0.13 mmol) in anhydr. DMF (1 ml) was stirred at rt for 5 h, then additional K_2_CO_3_ (85 mg, 0.62 mmol) was added, followed by additional iodomethane (100 µl, 1.61 mmol). The mixture was stirred at rt for a further 16 h. The mixture was diluted with ice water (5 ml) and extracted with EtOAc (2 × 5 ml). The combined organic fractions were washed with water, brine and dried (MgSO_4_) and concentrated under reduced pressure. The residue was suspended in CH_2_Cl_2_. Hot filtration removed the undissolved starting material, and the filtrate was concentrated under reduced pressure. Recrystallization of the residue (CH_2_Cl_2_/MeOH) afforded the methoxyindole **28** as yellow-orange crystals (14 mg, 64%), mp 164-168 ^o^C; δ_H_(500 MHz, CDCl_3_) 8.21 (1 H, d, *J*_4,5_ 8.7 Hz, H4), 7.10-7.24 (4 H, m, C_6_**H**_4_OCH_3_), 7.00 (1 H, dd, *J*_4,5_ 8.7, *J*_5,7_ 2.3 Hz, H5), 6.45 (1 H, d, *J*_5,7_ 2.3 Hz, H7), 3.92 (3 H, s, OCH_3_), 3.75 (3 H, s, OCH_3_), 2.64 (3 H, s, CH_3_); δ_C_(125 MHz, CDCl_3_)* 160.5 (1 C, C4'), 158.0 (1 C, C6), 142.3 (1 C, C2), 137.4 (1 C, C9), 129.3 (2 C, C2',6'), 127.7 (1 C, C1'), 127.6 (1 C, C3), 121.6 (1 C, C4), 115.4 (2 C, C3',5'), 115.3 (1 C, C8), 113.1 (1 C, C5), 95.1 (1 C, C7), 55.83, 55.82 (2 C, 2 × OCH_3_), 13.8 (1 C, CH_3_); HRMS (ESI^+^) *m/z* 313.1493 (C_17_H_17_N_2_O_4_ [M + Na]^+^ requires 313.1495).

*Carbon spectrum fully assigned using HSQC and HMBC, as the peak for the C3 carbon did not exhibit a signal in the 1-D ^13^C NMR spectrum.

**6-Benzyloxy1-(*p*-methoxyphenyl)-2-methyl-3-nitroindole (29)**

A mixture of benzyl bromide (20 µl, 0.16 mmol), **ID-8** (41 mg, 0.14 mmol), and K_2_CO_3_ (28 mg, 0.21 mmol) in anhydr. DMF (2 ml) was stirred at rt for 24 h. Additional K_2_CO_3_ (50 mg, 0.36 mmol) was added, followed by additional benzyl bromide (70 µl, 0.59 mmol) and the mixture was stirred at rt for a further 24 h. The mixture was diluted with ice water (5 ml) and extracted with EtOAc (2 × 5 ml). The combined organic fractions were washed with water, brine and dried (MgSO_4_) and concentrated under reduced pressure. The residue was suspended in CH_2_Cl_2_. Hot filtration of the solution removed the undissolved starting material, and the crude filtrate was concentrated under reduced pressure. Recrystallization of the residue (CH_2_Cl_2_/MeOH) afforded the benzyloxyindole **29** as dark yellow crystals (17 mg, 31%), mp 134-138 ^o^C; δ_H_(500 MHz, CDCl_3_) 8.22 (1 H, m, H7), 7.31-7.39 (5 H, m, C_6_H_5_), 7.08-7.23 (3 H, m, C_6_H_4_, H5), 6.55 (1 H, m, H4), 4.99 (2 H, s, PhC**H**_2_), 3.92 (3 H, s, CH_3_O), 2.65 (3 H, s, CH_3_); δ_C_(125 MHz, CDCl_3_) 182.9, 160.4, 157.0, 142.5, 137.2, 136.8, 129.3, 128.7, 128.2, 127.8, 127.6, 121.6, 115.5, 115.4, 113.6, 96.6 (20 C, Ar), 70.7 (1 C, CH_2_), 55.8, 13.8 (2 C, 2 × CH_3_); HRMS (ESI^+^) *m/z* 389.1493 (C_23_H_21_N_2_O_4_ [M + H]^+^ requires 389.1495).

**6-Acetoxy-1-(*p*-methoxyphenyl)-2-methyl-3-nitroindole (30)**

A solution of acetic anhydride (100 µl, 1.01 mmol), DMAP (1 mg, 0.01 mmol) and hydroxyindole **ID-8** (53 mg, 0.18 mmol) in pyridine (1.5 ml) was stirred at rt for 3 h. The resultant precipitate was collected by filtration and washed with pet. spirits. to afford acetoxyindole **30** as a pale yellow powder (25.3 mg, 42%), mp 199-201 ^o^C; δ_H_(500 MHz, CDCl_3_) 8.33 (1 H, m, H4), 7.08-7.27 (4 H, m, C_6_H_4_), 7.09 (1 H, m, H5), 6.78 (1 H, m, H7), 3.91 (3 H, s, OCH_3_), 2.68 (3 H, s, ArC**H**_3_), 2.26 (3 H, s, CO**C**H_3_); δ_C_ (125 MHz, CDCl_3_) 169.8 (1 C, C=O), 160.6 (1 C, C4'), 148.0 (1 C, C6), 143.7 (1 C, C2), 136.4 (1 C, C9), 129.3 (2 C, C2',6'), 127.5 (1 C, C3), 127.3 (1 C, C1'), 121.5 (1 C, C4), 119.1 (1 C, C8), 118.5 (1 C, C5), 115.5 (2 C, C3',5'), 104.6 (1 C, C7), 55.8 (1 C, OCH_3_), 21.2 (1 C, CO***C***H_3_), 13.8 (1 C, CH_3_); HRMS (ESI^+^) *m/z* 341.1130 (C_18_H_17_N_2_O_5_ [M + H]^+^ requires 341.1131).

**6-Hydroxy-1-(*p*-hydroxyphenyl)-2-methyl-3-nitroindole (45)**

AlCl_3_ (150 mg, 1.13 mmol) was added to a solution of NaI (30 mg, 0.20 mmol) and 6-hydroxyindole **ID-8** (40 mg, 0.134 mmol) in dry CH_2_Cl_2_ (2 ml) in an oven-dried flask fitted with a condenser. The reaction was refluxed overnight, and allowed to cool to rt. The solution was diluted with aq HCl (6 M, 2 ml), the CH_2_Cl_2_ was removed under pressure, and the precipitate was collected by filtration. The crude material was washed with aq 3 M HCl, followed by water, and purified by flash chromatography (20-80% EtOAc/pet. sp.). Recrystallization (EtOH/n-pentane) afforded the *N*-(*p*-hydroxyphenyl)indole **45** as yellow crystals (10.9 mg, 29%), mp 251-253 ^o^C; δ_H_(500 MHz, d_6_-MeOH) 8.00 (1 H, m, H4), 7.23-7.10 (4 H, m, C_6_H_4_), 6.86 (1 H, m, H5), 6.40 (1 H, m, H7), 2.61 (3 H, s, CH_3_); δ_C_ (125 MHz, d_6_-MeOH) 160.1, 156.7, 143.95, 139.1, 130.4, 128.1, 127.7, 122.0, 117.6, 115.4, 114.6, 97.9 (14 C, Ar), 13.7 (1 C, CH_3_); HRMS (ESI^+^) *m/z* 285.0869 (C_15_H_13_N_2_O_4_ [M + H]^+^requires 285.0870).

**2-(*m*-Methoxyphenylamino)-1-nitropropene (3)**

A solution of *p*-toluenesulfonic acid (150 mg, 0.87 mmol), *m*-anisidine (97 mg, 0.79 mmol) and nitroenamine **1** (124 mg, 0.95 mmol) in DMF (0.5 ml) was stirred at rt for 2 h. The mixture was diluted with water (5 ml) and extracted with EtOAc (3 × 5 ml) and the combined fractions were washed with and brine, dried (MgSO_4_) and concentrated under reduced pressure. The residue was purified by flash chromatography (10-30% EtOAc/pet. spirits) to afford *m*-methoxyphenylaminopropene **3** as an orange oil (112 mg, 68%); δ_H_(500 MHz, CDCl_3_) 11.54 (1 H, bs, NH), 7.31-7.34 (1 H, m, H5), 6.76-6.89 (2 H, m, H4,6), 6.71-6.72 (1 H, m, H2), 6.66 (1 H, s, C=CH), 3.83 (3 H, s, OCH_3_), 2.01 (3 H, s, CH_3_); δ_C_(125 MHz, CDCl_3_) 160.6, 156.2, 137.8, 130.5, 117.8, 113.2, 112.0, 111.5 (8 C, C=C,C_6_H_4_), 55.6 (1 C, OCH_3_), 18.2 (1 C, CH_3_); HRMS (ESI^+^) *m/z* 209.0928 (C_10_H_13_N_2_O_3_ [M + H]^+^ requires 209.0926).

**2-(*o*-Methoxyphenylamino)-1-nitropropene (4)**

A solution of *p*-toluenesulfonic acid (132 mg, 0.77 mmol), *o*-anisidine (260 µl, 2.30 mmol) and nitroenamine **1** (100 mg, 0.77 mmol) in DMF (40 µl) was stirred at rt for 2 h. The mixture was diluted with water (5 ml), the water layer was decanted, and EtOAc (1.5 ml) was added to the crude oil. A precipitate formed which was collected by filtration and washed with EtOAc to afford the *o*-methoxyphenylaminopropene **4** as yellow crystals (80 mg, 50%), mp 113-114 ^o^C; δ_H_(500 MHz, CDCl­_3_) 11.47 (1 H, bs, NH), 6.97-7.32 (4 H, m, C_6_H_4_), 6.69 (1 H, s, C=CH), 3.88 (3 H, s, OCH_3_), 1.98 (3 H, s, CH_3_); δ_C_(125 MHz, CDCl­_3_) 156.7, 153.5, 128.8, 126.2, 125.8, 120.8, 112.1, 111.8 (8 C, Ar), 55.9 (1 C, OCH_3_), 18.1 (1 C, CH_3_); HRMS (ESI^+^) *m/z* 209.0930 (C_10_H_13_N_2_O_3_ [M + H]^+^ requires 209.0926).

**6-Hydroxy-1-(*m*-methoxyphenyl)-2-methyl-3-nitroindole (48)**

A solution of nitroenamine **3** (112 mg, 0.54 mmol) and *p*-benzoquinone (58 mg, 0.54 mmol) in acetic acid (1 ml) and acetic anhydride (0.1 ml) was stirred at rt for 48 h. The resultant precipitate was collected by filtration and washed with acetic acid, then water. The crude material was recrystallized (AcOH) to afford hydroxyindole **48** as brown-yellow crystals (26 mg, 16%), mp 217-219 ^o^C (decomp.); δ_H_(400 MHz, CD_3_OD)* 8.02 (1 H, m, H4), 6.99-7.59 (4 H, m, C_6_H_4_), 6.87 (1 H, m, H5), 6.43 (1 H, m, H7), 3.87 (3 H, s, OCH_3_), 2.64 (3 H, s, ArC**H**_3_); δ_C_(125 MHz, CD_3_OD) 162.5 (1 C, C3'), 156.8 (1 C, C6), 143.3 (1 C, C2), 138.6 (1 C, C9), 137.6 (1 C, C1'), 132.0 (1 C, C5'), 128.4 (1 C, C3), 122.1 (1 C, C4), 121.2 (1 C, C6'), 116.7 (1 C, C4'), 115.4 (1 C, C8), 114.8 (1 C, C5), 114.7 (1 C, C2'), 97.8 (1 C, C7), 56.2 (1 C, OCH_3_), 13.8 (1 C, Ar**C**H_3_); HRMS (ESI^+^) *m/z* 299.1038 (C_16_H_15_N_2_O_4_ [M + H]^+^ requires 299.1026).

***** Carbon spectrum fully assigned using HSQC and HMBC (due to extremely weak signal for C3 carbon in 1D ^13^C NMR spectrum).

**6-Hydroxy-1-(*o*-methoxyphenyl)-2-methyl-3-nitroindole (49)**

A solution of nitroenamine **4** (119 mg, 0.57 mmol) and *p*-benzoquinone (62 mg,0.57 mmol) in acetic acid (1.1 ml) and acetic anhydride (0.11 ml) was stirred at rt for 48 h. The solution was diluted with water (5 ml) and extracted with CH_2_Cl_2_ (3 × 5 ml). The combined organic fractions were washed with sat. aq. NaHCO_3_ and brine, dried (MgSO_4_), and concentrated under reduced pressure. The crude product was purified by flash chromatography (2% MeOH in CH_2_Cl_2_) followed by recrystallization (CHCl_3_) to afford hydroxyindole **49** as yellow crystals (31 mg, 18%), mp 200-203 ^o^C; δ_H_(500 MHz, CD_3_OD); 8.00 (1 H, m, H4), 7.19-7.64 (4 H, m, C_6_H_4_), 6.86 (1 H, m, H5), 6.27 (1 H, m, H7), 3.79 (3 H, s, OCH_3_), 2.54 (3 H, s, CH_3_); δ_C_(125 MHz, CD_3_OD) 157.1, 156.7, 144.5, 138.6, 132.8, 130.8, 124.6, 122.4, 122.0, 115.5, 114.5, 113.9, 97.67, 97.66 (14 C, Ar), 56.4 (1 C, OCH_3_), 13.4 (1 C, CH_3_); HRMS (ESI^+^) *m/z* 299.1035 (C_16_H_15_N_2_O_4_ [M + H]^+^ requires 299.1026).

**2-Methylindole (5)**

A solution of phenylhydrazine (500 mg, 4.62 mmol) in acetone (373 µL, 5.08 mmol) was heated at 80 ^o^C for 2 h. The solution was concentrated under reduced pressure and azeotroped with toluene to remove excess water, and dried at 0.05 mmHg for 2 h. The resultant oil was added to freshly fused ZnCl_2_ and the mixture was stirred at 160 ^o^C overnight. The mixture was quenched with water and extracted with CH_2_Cl_2_. The combined extracts were washed with brine, dried (MgSO_4_) and concentrated under reduced pressure. The crude product was purified by flash chromatography (15-100% CH_2_Cl_2_/pet. spirits) to afford 2-methylindole (**5**) as an off-white powder (383 mg, 63%); mp 57-59 ^o^C (lit.[^4^](#_ENREF_4) 58-59 ^o^C); δ_H_(600 MHz, CDCl_3_) 7.80 (1 H, bs, NH); 7.08-7.54 (4 H, m, H4,5,6,7), 6.24 (1 H, s, H3), 2.45 (3H, s, CH_3_); HRMS (ESI^+^) *m/z* 132.0810 (C_9_H_9_N [M + H]^+^ requires 132.0808).

***N*-(*p*-Methoxyphenyl)-2-methylindole (6)**

A mixture of *N,N'*-dimethylethylenediamine (94 µl, 0.87 mmol), 2-methylindole (**5**) (285 mg, 2.17 mmol), 4-iodoanisole (560 mg, 2.39 mmol) and 1,4-dioxane (0.5 ml) in toluene (1.5 ml) was cooled to -196 ^o^C and the flask was evacuated, then backfilled with nitrogen and returned to rt. The freeze-pump-thaw cycle was repeated three times. CuI (83 mg, 0.43 mmol) and ground K_3_PO_4_.H_2_O (1.00 g, 4.34 mmol) was added to the flask, and two more freeze-pump-thaw cycles were performed. The mixture was refluxed overnight. The solution was cooled to rt, filtered through a pad of Celite (CH_2_Cl_2_), and purified by column chromatography (0-40% EtOAc/pet. sp.) to afford indole **6** as clear crystals (171 mg, 33%), mp 71-72.6 ^o^C (EtOAc/pet. sp.); δ_H_(400 MHz, CDCl_3_) 7.57-7.03 (8 H, m, H4,5,6,7,C_6_H_4_), 6.38 (1 H, s, H3), 3.90 (3 H, s, OCH_3_), 2.28 (3 H, s, CH_3_); δ_C_(125 MHz, CDCl_3_) 159.1, 138.6, 137.5, 130.8, 129.3, 128.2, 121.0, 120.0, 119.6, 114.7, 110.1, 100.9 (14 C, Ar), 55.6 (1 C, OCH_3_), 13.4 (1 C, CH_3_); HRMS (ESI^+^) *m/z* 238.1225 (C_16_H_15_NO [M + H]^+^ requires 238.1226).

**1-(*p*-Methoxyphenyl)-2-methyl-3-nitroindole (38)**

A cooled solution of 70% HNO_3_ (aq.) (66 µl, 1.107 mmol) in acetic anhydride (821 µl) was added dropwise to a solution of indole **6** (99 mg, 0.369 mmol) in acetic anhydride (4.9 ml) at -40 ^o^C. The mixture was stirred for a further 30 min, maintaining the temperature between -50 ^o^C and -40 ^o^C. The resultant suspension was diluted with ice and extracted with EtOAc (20 ml). The organic phase was washed with sat. aq. NaHCO_3_ (3 × 10 ml), water (2 × 5 ml), brine (2 ml) and dried (Na_2_SO_4_). The solution was concentrated to a volume of 5 ml under reduced pressure, and diluted with toluene (2 ml). Further reduction of volume under reduced pressure resulted in precipitation, and the precipitate was collected and washed with toluene to afford the nitroindole **38** as yellow crystals (48.4 mg, 46%), mp 214-217 ^o^C; δ_H_(500 MHz, CDCl_3_) 8.34-8.36 (1 H, m, Ar), 7.36-7.40 (1H, m, Ar), 7.09-7.27 (5 H, m, Ar,C_6_H_4_), 7.01-7.03 (1 H, m, Ar), 3.94 (3 H, s, OCH_3_), 2.69 (3 H, s, CH_3_); δ_C_ (125 MHz, CDCl_3_) 160.5 (1 C, C=O), 143.1 (1 C, C2), 136.4 (1 C, C9), 129.3 (2 C, C2',6'), 127.7 (1 C, C1'), 127.6 (1 C, C3), 124.4 (1 C, C6), 124.3 (1 C, C5), 121.2 (1 C, C8), 120.7 (1 C, C4), 115.4 (2 C, C3',5'), 111.2 (1 C, C7), 55.8 (1 C, OCH_3_), 13.82 (1 C, CH_3_); HRMS (ESI^+^) *m/z* 283.1080 (C_16_H_14_N_2_O_3_ [M + H]^+^requires 283.1077).

**References**

1 A. B. Pangborn, M. A. Giardello, R. H. Grubbs, R. K. Rosen and F. J. Timmers, *Organometallics*, 1996, **15**, 1518.

2 T. Severin, D. Scheel and P. Adhikary, *Chem. Ber.*, 1969, **102**, 2966.

3 V. M. Lyubchanskaya, L. M. Alekseeva and V. G. Granik, *Khim. Geter. Soed.*, 1992, **1**, 40.

4 A. E. Wendlandt and S. S. Stahl, *J. Am. Chem. Soc.*, 2014, **136**, 506.
