# Supplementary material for: Inhibition of DYRK1A disrupts neural lineage specificationin human pluripotent stem cells
Source: eLife. 2017 Sep 8;6:e24502. doi: 10.7554/eLife.24502 (PMC5656431; doi:10.7554/eLife.24502)
Supplement: Supplementary file 3. — Table shows IC50 (nM) or percentage inhibition values for compounds against a series of CMGC kinase family members. Values are from this study (ID-8, see Materials and methods) or the literature cited. [file elife-24502-supp3.docx]

**Supplementary File 3. Kinase inhibition of ID-8 related to other known DYRK1A inhibitors**

| **Inhibitor** | **IC_50_ (nM) / %inhibition** | | | | | | | | | |
| --- | --- | --- | --- | --- | --- | --- | --- | --- | --- | --- |
|  | **CLK1** | **CLK2** | **CLK3** | **CLK4** | **DYRK1A** | **DYRK1B** | **DYRK 2** | **DYRK 4** | **GSK3α** | **GSK3β** |
| **ID-8**^1^**^,a^** | 4200 | >10,000 | >10,000 | 440 | 78 | 54 | >10,000 | >10,000 | 380 | 450 |
| **Harmine**^2^**^,b^** | 95%^3^ | 91%^3^ | 70%^3^ | - | 33 | 166 | 1931 | 79750 | - | Low inhibition^4^ |
| **TG003** | 20^5,c^ | 200^5,c^ | >1000^5,c^ | 15^5,c^ | 930^3,d^ | 1750^3,d^ | - | - | - | - |
| **INDY**^3^**^,d^** | 99% | 88% | 70% | 100% | 240 | 230 | 97% | 87% | 70% | 52% |
| **ProINDY**^3^ | - | - | - | - | 26% | - | - | - | - | - |
| **NC037**^4^**^,e^** | 59 | 1902 | 6936 | 39 | 62 | 697 | - | - | - | - |
| **NC981**^4^**^,e^** | 20 | 186 | 1924 | 11 | 14 | 25 | - | - | - | - |
| **NC3104^4,e^** | 173 | 584 | 435 | 70 | 17 | 83 | - | - | - | - |
| **L415^,f^** | 71 | 720 | >10,000 | 64 | 60 | 44 | 73 | 520 | 210 | 380 |
| 1. (Miyabayashi et al., 2008)  2. (Gockler et al., 2009)  3. (Ogawa et al., 2010)  4. (Rosenthal et al., 2011)  5. (Tahtouh et al., 2012)  ^a^ Our data: Maximum inhibitor concentration = 30 µM. [ATP] = 10µM. [γ-^33^P]ATP = 10 µCi/µl. Enzyme concentration = 0.2 - 50 nM. Substrate concentration = 20 µM.  ^b^ Maximum inhibitor concentration = 100 µM. [ATP] = 100 µM. [γ-^33^P]ATP = 0.6 µCi. Enzyme concentration = 0.4 - 5 mU. Substrate concentration = 100 µM.  ^c^ Maximum inhibitor concentration = NR. [ATP] = 1-20 µM. [γ-^32^P]ATP = 1 µCi. Enzyme concentration = 0.0025-0.025 g/l (0.1-1 µg in 40 µl). Substrate concentration = 20 µM.  ^d^ For IC_50_ values: Maximum inhibitor concentration = 40 µM. [ATP] = 10µM. [γ-^32^P]ATP = 4 µCi. Enzyme concentration = 0.004-0.04 g/l (0.1-1 µg in 25 µl). Substrate concentration = 0.025 g/l (1 µg in 40 µl). For % inhibition: [INDY] = 10µM  ^e^ Maximum inhibitor concentration = 50 µM. [ATP] = 10 µM. [γ-^33^P]ATP = NR. Enzyme concentration = NR. Substrate concentration = 20 µM.  ^f^ Maximum inhibitor concentration = 10 µM. [ATP] = 15 µM. [γ-^33^P]ATP = NR. Enzyme concentration = NR. Substrate concentration = 1µg/ 30 µl. | | | | | | | | | | |
